# A case report of an ampullary tumor presenting with spontaneous perforation of an aberrant bile duct and treated with total laparoscopic pancreaticoduodenectomy

**DOI:** 10.1186/1477-7819-10-142

**Published:** 2012-07-12

**Authors:** Mehmet Kaplan

**Affiliations:** 1 Department of General Surgery, Medical Park Gaziantep Hospital, Gaziantep, Turkey

**Keywords:** Bile peritonitis, Aberrant bile duct, Spontaneous bile duct perforation, Laparoscopic pancreaticoduodenectomy, Whipple procedure, Pancreatic neoplasm, Duodenal neoplasm, Common bile duct, Treatment, Surgery, Laparoscopy

## Abstract

**Background:**

This case report discusses a patient who presented with bile peritonitis due to spontaneous perforation of an aberrant bile duct that originated in the triangular ligament of the liver. It was associated with an ampullary tumor and treated with total laparoscopic pancreaticoduodenectomy (TLPD).

**Case report:**

A 58-year-old male patient was admitted to the emergency department of Medical Park Gaziantep Hospital in September 2009 with acute abdominal findings. He underwent an urgent laparoscopy, and, interestingly, bile peritonitis due to the rupture of an aberrant bile duct in the triangular ligament was noted. After laparoscopic treatment of the acute conditions, the follow-up examinations of the patient showed the finding of obstructive jaundice. Endoscopic retrograde cholangio-pancreatography revealed a 1-cm polypoid mass located at the ampulla of Vater (duodenal papilla) with possible extension to the ampullary sphincter. A stent was inserted for temporary biliary drainage, and subsequent endoscopic biopsy showed the pathological finding of adenocarcinoma.

After waiting for a 1-month period for the peritonitis to heal, the patient underwent pylorus-preserving TLPD and was discharged without any major complications on postoperative day 7.

**Conclusion:**

In patients with bile peritonitis, it should be considered that the localization of the perforation may be in an aberrant bile duct localized at the triangular ligament and the etiology may be associated with an obstructing periampullary tumor. Laparoscopic pancreaticoduodenectomy is a feasible operative procedure in carefully selected patients. This technique can achieve adequate margins and follows oncological principles. Randomized comparative studies are needed to establish the superiority of minimally invasive surgery over traditional open surgery.

## Background

Spontaneous bile duct perforation (SBDP) is rarely seen. It usually develops in newborns and children because of congenital biliary tract abnormalities [1]. It is much rarer in adults. When seen, the etiological factors in almost all adult cases are bile stones, and the site of perforation is commonly extrahepatic [2,3]. A case of bile peritonitis due to the spontaneous rupture of an aberrant bile duct has not yet been reported, and the literature is limited to reports of only a few cases of SBDP caused by tumors [4,5]. A case of bile peritonitis as a consequence of perforation of an aberrant bile duct, associated with an ampullary tumor, has never been previously described in the literature.

In the past 15 years, significant advances in laparoscopic surgical skills and techniques combined with dramatic advances in laparoscopic technology have encouraged the application of laparoscopy to the evaluation and treatment of solid organs including the pancreas. Because of the complexity of the procedure, long operating time and higher complication rate, laparoscopic pancreaticoduodenectomy has not gained worldwide popularity. In addition to the biliary pathology, which caused the interesting presentation of the patient, this case report also describes a TLPD for a tumor of the ampulla of Vater with a successful outcome, representing the first description of this laparoscopic procedure in Turkey.

## Case report

A 58-year-old male patient, without any history of trauma or important previous symptoms, was admitted to the emergency department of Medical Park Gaziantep Hospital on September 2009 with the complaint of sudden onset of severe abdominal pain. Physical examination revealed signs of an acute abdomen. Laboratory tests showed leukocytosis, but the renal and liver function tests, and serum amylase and lipase levels were normal. Chest and abdominal X-ray revealed no free air in the abdomen. An ultrasound examination revealed large amounts of free fluid in the abdomen, and gallbladder hydrops was identified. However, a finding of a gallbladder and common bile duct stone was not reported.

**Figure 1:**
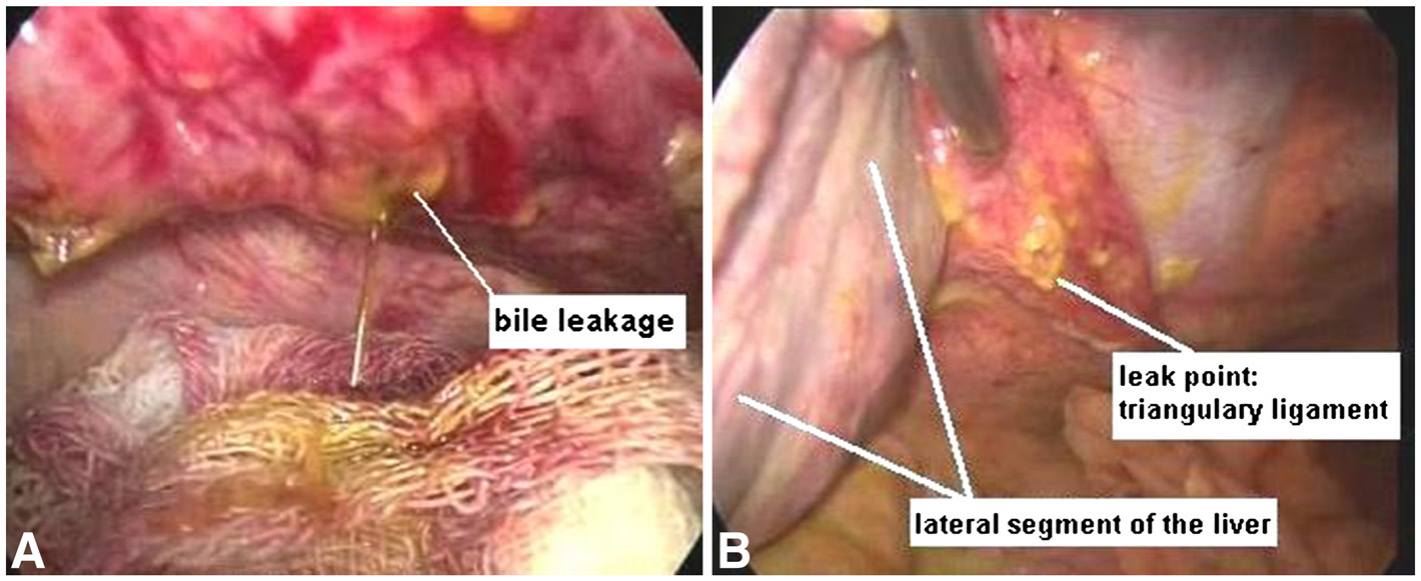
(**A) Bile leakage from the triangular ligament.** (**B**) Laparoscopic findings of bile leakage.

Because of these findings, the patient underwent diagnostic laparoscopy with the prediagnosis of hollow organ perforation. However, exploration revealed bile peritonitis, and approximately 1 l of bile was aspirated. The gallbladder, pylorus, duodenum and common bile duct, the possible regions of bile leakage, were investigated, but there was no perforation. Continuous accumulation of bile in the left subphrenic region focused the exploration on this region. Interestingly, bile leakage was detected at the left-end part of the triangular ligament. It was understood that the bile peritonitis was associated with spontaneous perforation of an aberrant bile duct in the appendix fibrosa hepatis (Figure 1A, B). Leakage was controlled with a few intracorporeal sutures (Additional file 1: Video 1).

**Figure 2:**
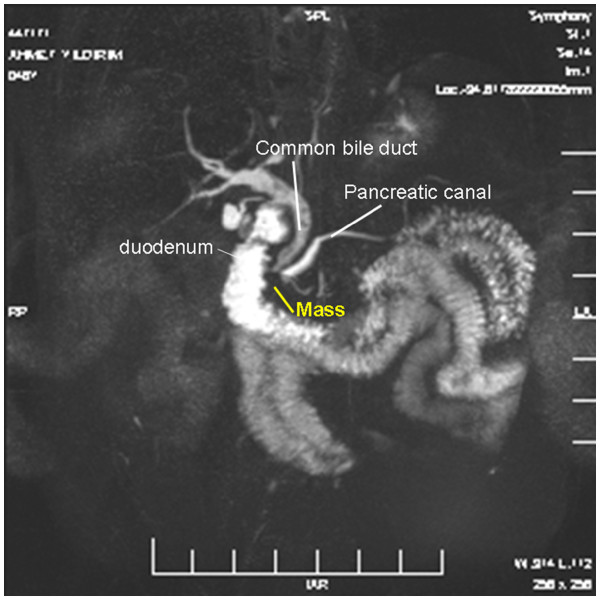
MRCP-detected mass.

The postoperative period was uneventful. However, close examination of the patient during follow-up revealed obstructive jaundice. MRCP detected a 1-cm tumor mass in the region of the ampulla of Vater and common bile duct dilatation. However, there were no biliary tract stones (Figure 2) (Additional file 2: Video 2). These findings revealed aberrant bile duct perforation developed secondarily to ampullary tumor mass effect. ERCP was performed, and a plastic stent was inserted for drainage. The biopsy, taken during the procedure, was reported as adenocarcinoma.

Curative surgery was planned within 1 month after the improvement of the peritoneal findings. Operability and tumor stage were assessed according to the preoperative radiological imaging tests, which demonstrated a tumor limited to the ampulla of Vater with no any evidence of regional lymph node enlargement or distant metastasis. According to the currently accepted American Joint Committee on Cancer staging system for ampullary carcinoma, the preoperative tumor stage was T1N0M0 and stage I.

Among different options for curative surgery, TLPD was preferred. The reasons and criteria for choosing laparoscopic instead of conventional open surgery were as follows: (1) the early stage of the tumor, (2) the absence of a systemic disease that contraindicated long-term general anesthesia, (3) the absence of a pathology that absolutely required avoiding laparoscopic surgery, (4) the patient had a very suitable body mass index of 24 kg/m^2^ for laparoscopic surgery, (5) the presence of a surgeon who had sufficient experience in laparoscopy, having carried out more than 100 intracorporeal gastrointestinal and biliary anastomoses (both in humans and swine) and more than 500 advanced laparoscopic surgeries including distal pancreatic resections, enucleations and extrahepatic biliary tract surgeries, and (6) the preference of the patient.

This patient was the first in whom a TLPD was attempted. Therefore, the possible advantages and complications of this new surgical procedure and, at any stage, the possibility of conversion to traditional open surgery were described in detail to the patient. Thereafter, the patient gave his written informed consent, choosing this method instead of conventional surgery. The local ethics review board approved the procedure.

**Figure 3:**
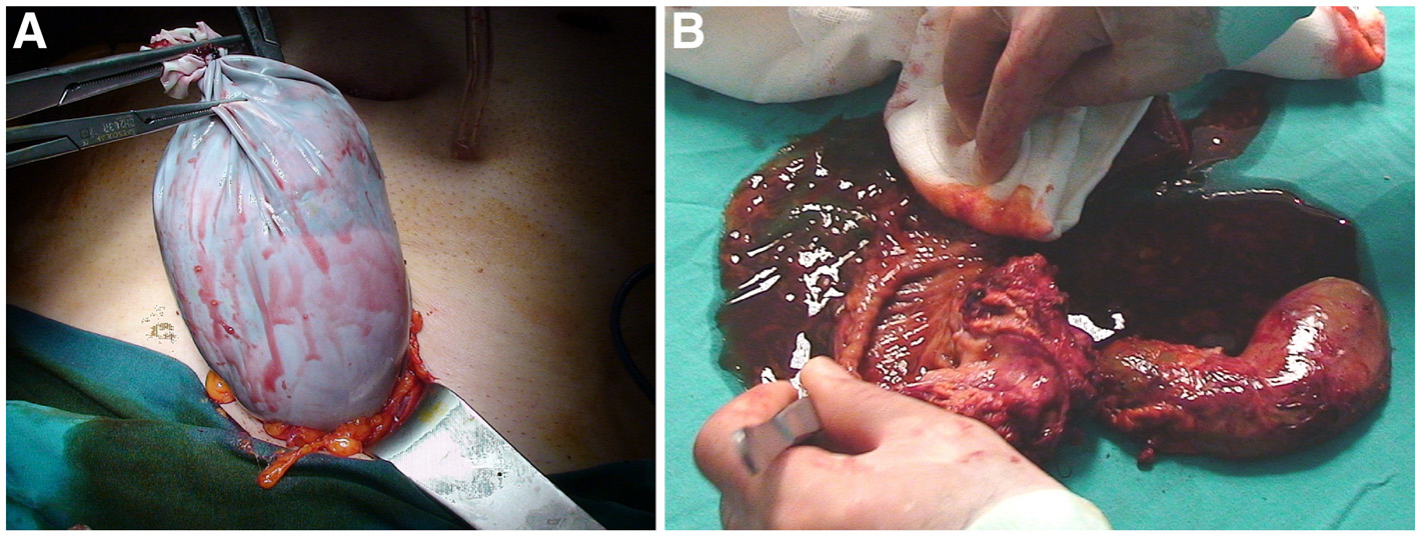
(**A) Specimen removal from a suprapubic incision.** (**B**) Retrieved specimen.

In the second surgery, the patient underwent complete laparoscopic pyloric protective pancreaticoduodenectomy. A total of five trocars were used. According to the nature of the tissue, the combination of an ultrasonic dissector, vessel sealing device, and monopolar hook coagulator was used in all dissections. The duodenum and jejunum were transected with an endo-GIA stapler. The neck of the pancreas was divided using an ultrasonic dissector. All anastomoses were performed intracorporeally, and in all of them single-filament absorbable 4/0 polydioxanone sutures were used. Because of the technical ease, pancreaticogastrostomy was preferred for pancreatic reconstruction. Then, a single-layer hepaticojejunostomy and finally a double-layer duodenojejunostomy anastomosis was performed on the same jejunal loop. The specimen was removed through the 6-cm suprapubic incision in accordance with oncological principles (Figure 3A, B) (Additional file 3: Video 3, Additional file 4: Video 4, Additional file 5: Video 5 and Additional file 6: Video 6). There were no complications and no need for blood transfusions. Operative time was 510 min.

Pathological evaluation revealed an exposed protruding-type and well-differentiated adenocarcinoma macroscopically and microscopically, respectively, with negative surgical margins, with 14 lymph nodes retrieved and no tumor invasion. The patient was started on oral nutrition on day 5 and discharged on postoperative day 7. On follow-up examination after 6, 12, and 24 months, there was no evidence of recurrence or metastasis of the tumor on abdominal and thoracic CT, and the blood test showed only mild hyperglycemia.

## Discussion

It has been reported that all kinds of pathologies that increase the intraductal biliary tract pressure in adults, such as stasis or dilatation of the bile ducts (distal obstruction of the main bile duct, disease or spasm of the sphincter of Oddi, pancreatitis), abnormalities of the bile duct wall (diverticula), infection, connective tissue diseases, or ischemia, can lead to spontaneous bile duct perforation [2,3,6-8]. When compared with a sudden pressure increase caused by the stones, slow-growing tumors, which slowly increase the intraductal pressure, are thought to be less likely to cause rupture of the bile duct [3]. This may be why only a few cases in the literature are limited to tumor-related SBDP [4,5]. In the patient presented here, the etiological cause of bile peritonitis was an ampullary tumor. It has been proposed that the sudden decrease in obstructed bile pressure after perforation may have led to rapid improvement in the liver function tests [9]. The normal liver function test results of our patient at the emergency department at the time of application supports this view.

In patients with SBDP, the puncture sites are almost always extrahepatic bile ducts, and the common bile duct is the most commonly involved [3]. An aberrant bile duct, in the left triangular ligament of the liver in our patient, has been perforated because of increased bile pressure secondary to a tumor itself. Spontaneous perforation of such a channel has never been reported in the literature so far. The presence of aberrant bile ducts (vasa aberrantia) has been reported previously. However, there have been few studies on their clinical significance, and they are observed much more often than anticipated. More than 30% of them were only recognized in the gallbladder bed. In addition to being on the surface of the liver in a fibrous sheath or in the left triangular ligament, they may also be seen as a separate channel [10].

It has been suggested that, particularly because of the main bile duct stenosis or obstruction, aberrant bile ducts could have become functionally active [11]. Furthermore, these ducts may be responsible for some of the cases of biliary peritonitis; thus, they should be well known by surgeons. Recently, Kekis and colleagues [12] identified aberrant bile ducts in the left triangular ligament in 16% of cadaver livers and in 12% of *in-situ* livers studied. They also cut this ligament during surgery and detected bile leakage, thus emphasizing the clinical importance of these ducts in appendix fibrosa hepatis. Similar findings have been confirmed by other authors, and aberrant bile peritonitis developing from damage to the channels in this area has been described [13].

As a result of the rapid development of laparoscopic surgery treatment of many pancreato-biliary pathologies by minimal invasive surgery, as in our patient, has become possible. Laparoscopic resection of the pancreas has been applied in a very different disease group, such as chronic pancreatitis, pancreatic trauma, hyperinsulinism and cystic lesions. However, the issue of proximal pancreatectomy for cancer surgery is still controversial. Although laparoscopic pancreaticoduodenectomy is technically feasible, completion of laparoscopic reconstructions after resection has not yet become general practice. Therefore, in a significant proportion of reported cases, the reconstructions were carried out through a mini-laparotomy incision [14-17]. On the other hand, the recently published largest series with 75 cases revealed in all patients that reconstructions can be done completely laparoscopically with no need of laparotomy or converting to open surgery [18].

Many authors believe that the distal pancreatic resection with the laparoscope is entirely possible and effective; however, because pancreaticoduodenectomy requires a long operative time, can have multiple complications and has a high death rate, several authors don’t advocate doing this operation with a laparoscope. On the other hand, the general experience with minimally invasive abdominal surgery suggests that the benefits of laparoscopic pancreatic surgery may include decreased incisional complications, less postoperative pain, faster return of digestive function, faster return to normal activities, development of fewer intra-abdominal adhesions, diminished procedure-related inflammatory responses and alterations in host immune function, which may be one of the important advantages for the cancer patients, while providing enhanced vision and magnification of anatomic structures. I think no significant difference exists between laparotomy and laparoscopy concerning mortality, morbidity and postoperative recovery; however, laparoscopy causes less pain and only requires a small incision.

## Conclusions

This case report shows that in patients with spontaneous bile peritonitis, even though rare, it should be considered that localization of the leak may be in an aberrant bile duct localized at triangular ligament of the liver and the etiology may be associated with an obstructing periampullary tumor.

This case report also supports the others showing that, in selected cases, TLPD is feasible. However, in order to achieve results comparable to open surgery and to become a more commonly performed surgery, development of some special hand tools and automated systems for biliary and pancreatic anastomosis, as well as a greater number of randomized comparative clinical studies are needed.

## Consent

Written informed consent was obtained from the patient for publication of this case report and any accompanying images. A copy of the written consent is available for review from the Editor-in-Chief of this journal.

## Endnotes

This study was presented as a video session at the 17th National Surgery Congress (Ulusal Cerrahi Kongresi), 26–29 May 2010, Sheraton Hotel, Ankara, Turkey.

## Competing interests

The author declare that has no competing interests.

## Supplementary Material

Additional file 1 **Video 1.** shows diagnostic laparoscopy and bile leak detected at the triangulary ligament of the liver. Click here for file

Additional file 2 **Video 2.** MRCP imaging series shows tumor localization. Click here for file

Additional file 3 **Video 3.** show TLPD technique performed in the patient. Click here for file

Additional file 4 **Video 4.** show TLPD technique performed in the patient. Click here for file

Additional file 5 **Video 5.** show TLPD technique performed in the patient. Click here for file

Additional file 6 **Video 6.** show TLPD technique performed in the patient. Click here for file
